# Association between leisure-time activities and school failure in adolescents: The 1993 Birth Cohort

**DOI:** 10.1371/journal.pone.0205793

**Published:** 2018-11-07

**Authors:** Fernando C. Wehrmeister, Romina Buffarini, Andrea Wendt, Caroline dos Santos Costa, Rosália Garcia Neves, Thaynã Ramos Flores, Juarez Lopes, Helen Gonçalves, Ana Maria Menezes

**Affiliations:** 1 Post-Graduate Program in Epidemiology, Federal University of Pelotas, Pelotas, Brazil; 2 Sul-rio-grandense Federal Institute, Pelotas, Department of Languages, Pelotas, Brazil; University of Pennsylvania, UNITED STATES

## Abstract

**Aim:**

To evaluate the relationship between leisure-time activities at 11 years old and the incidence of school failures from 11 to 15 years in adolescents.

**Methods:**

The sample comprised 4,090 adolescents from the 1993 Pelotas Birth Cohort, Brazil. The outcome was measured as the number of school failures from 11 to 15 years, based on reported information from cohort participants and their parents. The exposures were collected at 11 years old, as follows: reading; meeting friends; talking to parents; and dating.

**Results:**

In the group from 11 to 15 years old, 53.3% failed at school at least once. Meeting friends 4–7 times/week (RR = 1.15) and dating 1–3 times/week (RR = 1.22) were associated with high risk for school failure. Reading showed an inverse relationship with school failures (1–3 times/week RR = 0.83; 4–7 times/week RR = 0.71). Reading at least once a week could prevent around 16% of school failures.

**Conclusion:**

The context in which adolescents are inserted plays a relevant role in school performance. Understanding these factors may help to propose actions to reduce school failure rates even further.

## Introduction

Education is a social process acting as a developmental factor for the country, promoting citizenship and reducing socioeconomic inequalities [1]. To ensure its quality is an important challenge as far as public policies are concerned^1^. The high rates of failure at school in Brazil, which represent a historical problem, are highlighted among the many difficulties faced by the educational process in the country [2,3,4].

According to a series of historical school census made available by the *Instituto Brasileiro de Geografia e Estatística* [5], failure rates at the primary school level have been stable since 1999 and 2010 (10.4% a 10.3%). On the other hand, this rate has climbed from 7.2% to 12.5% in secondary schools for the same period [5]. A growing body of evidence shows that the rise in school failure rates in the last year of primary school may bring about a higher risk for school dropouts and therefore discontinue adolescents’ school life [6,7,8].

Failure and school dropouts have been associated to biological, psychological, environmental and family aspects [1]. In a broader context, life conditions such as socioeconomic, housing, sanitation facilities, health and nutrition represent possible school performance determinants for children and adolescents [1]. More specifically, the lack of communication between adolescents and parents, alcohol and illegal drugs use in addition to problems regarding the adolescent’s mental health are described in the literature as harmful factors regarding school performance [9,10]. Therefore, unfavourable family and socioenvironmental conditions would result in lower learning ability and higher probability of successive failure along the students’ lives, leading to school dropout and underemployment, making social inequalities broader [1].

Facing a scenario of great diversity regarding school failure determinants, it is likely that activities carried out by adolescents at leisure-time may influence school life. Not only an excess of physical activity practice [11] but also lack of it [12], for instance, have been suggested as risk factors for school failure while reading habits are considered protective ones [13]. Furthermore, some authors have shown that good relations with friends and family have a positive impact on the adolescents’ academic performance [14,15]. In addition, there is a direct relationship with consumption of alcoholic beverages, which may indirectly interfere with school performance [16,17]. Despite the importance of the theme, studies associating leisure-time activities (reading, social relationship and parental relationship at leisure-time) with school failure are scarce, especially with a cohort design. Therefore, the aim of this study is to assess the relationship between leisure-time activities in early adolescence and the incidence of school failure in a Brazilian birth cohort.

## Methods

All alive births whose mothers lived in the urban area in Pelotas and who delivered their babies between the 1st of January and the 31st of December 1993 were invited to participate in this birth cohort. A questionnaire regarding socioeconomic, demographic and behavioural aspects was carried out in a perinatal study. In addition, anthropometric measures were taken for mothers and babies. Along the following years, evaluations were carried out and either subsamples or all the participants were invited to answer new questionnaires and undergo anthropometric assessment among other measures and exams. Further information regarding the methodology used in the cohort study is described in other publications [18,19].

Data used in the present study come from the 2004/5 and 2008 follow-ups. In these two occasions, all members of the cohort were located and invited to participate in the evaluation. Retention rates (deaths + interviewed cohort members) were 87.5% and 85.7% from the original cohort at 11 and 15 years respectively [18,19].

The outcome was defined as the number of school failures (corresponding to a full school year) from 11 to 15 years. At the 11 year-old follow-up, the question about school failure was answered by the adolescent’s mother or guardian, using the following question: “*Has <name of the adolescent > ever failed at school*?”. If the answer was positive, the mother or guardian was also questioned about how many times the adolescent had failed. At the 15 year-old follow-up, the number of school failures was answered by the adolescent himself/herself using the following question *“Have you ever failed at school*?”. If the answer was positive, they would be questioned about how many times without specifying how many times in each grade. Before both visits, a pilot study with a sample similar to the cohort, in terms of socioeconomic position, age and sex was conducted. This procedure aimed to ensure internal validity for the all the questions, including those about school failures. The questions were adapted according to the participants’ understanding.

As both questions at 11 and 15 years follow-up asked for the number of failures during all life, the total number of school failures between 11 and 15 years was calculated by subtracting the number of failures at 11 years from the number of failures at 15 years. The adolescents who had never failed at school up to 15 years of age and those who only failed before 11 years were classified as not having failed in this period. The number of school failures varied from zero to five times and were grouped into categories from four to five times for having a reduced number of observations (n = 33 and n = 10, respectively).

The exposure variables in this study, answered by the 11-year old adolescents, were the following leisure-time activities; 1) reading magazines, newspapers or books; 2) meeting friends; 3) talking with parents; and 4) dating. The adolescents were questioned about how many days a week these activities were carried out. For a detailed description of leisure-time activities according to the number of failure outcome, the exposure variables were categorized into: “never during the week”, “from once to three times a week” and “four or more times a week”. Exposures were dichotomized in “never during the week” and “from once to seven days a week” for the analysis of attributable risk. The variables which were used as possible confounding factors, related to the perinatal follow-up were: sex (male/female), family income (minimum wages), self-reported skin colour of the adolescent, analysed in the same way it was collected (white, black, brown, yellow and indigenous) [20], mother’s schooling years (completed years), working outside the home (yes/no) and mental health using *Strengths and Difficulties Questionnaire* (SDQ) applied to mothers in order to identify mental health problems in adolescents [21]. The number of failures before age 11 was also used as a confounder variable.

Initially, a descriptive analysis was carried out, showing the absolute number and the frequency of the category variables, in addition to median and interquartile range of continuous variables. An initial analysis presented the average number of school failures according to the three exposure categories (never during the week /from once to three/four or more times a week).

In order to investigate the association between the practice of leisure-time activities and the number of school failures at 11 and 15 years, crude and adjusted analyses were conducted using Poisson regression (for discrete outcomes) with adjustment for the observed over-dispersion of the outcome (data not shown) to estimate the incidence rate ratio of school failures, expressed as relative risk (RR) in the tables. The associations with p-value<0,05 were considered significant.

Moreover, the preventable fraction [FP = % exposure*(1-RR)**/** RR+ %exposure *(1-RR)] and etiological fraction [FE = %exposure*(RR-1) / 1+ %exposure*(RR-1)] from exposure related to school failure were presented [22], for protective and risk factors, respectively. This analysis has allowed us to understand the excess of school failures related to each of the assessed leisure-time activities.

In addition, a cluster analysis was carried out to verify the frequency of the co-occurrence of the exposure variables. In this analysis, the cluster takes place when the observed prevalence from a combination of behaviours in the sample is higher than the expected prevalence for the same combination^23^. The expected prevalence for a certain combination is calculated based on multiplying the individual probabilities of each behaviour considering its observed occurrence [23,24].

Afterwards, activities which presented a RR higher than one in the crude analysis were combined into one variable and those which presented a RR lower than one into other. These two variables have the following categories: showing no behaviour, only one behaviour and showing two behaviours at the same time, regardless the type. The first category was used as a reference for both variables in a Poisson regression model, similarly to the analysis previously described.

All analyses were conducted using Stata® 12.1 (StataCorp. CollegeStation USA). All visits of the 1993 Pelotas birth cohort were approved by the Institutional Review Board at the Medicine School from the Federal University of Pelotas. Informed consent was given by parents’ participants. The authors did not have access to identification of subjects in any phase of the study. The collected data are strictly used for research purposes. The authors declare no conflicts of interest.

## Results

From the 5,249 members of the original cohort, 4,452 were interviewed at 11 years and 4,325 at 15 years. In the current analysis, 4,090 adolescents showed data related to the assessed outcome (78% of the original cohort). From these individuals, 51.5% were male, 64.3% self-reported having white skin and the majority worked outside the home at 11 years (95.6%). The family income median was 2.6 minimum wages (January minimum wage/1993≅$100,00), while mother-schooling median was six complete years. The prevalence of failing at least once between 11 and 15 years was 53.3% and the median was 1 (Table 1). S1 Table shows the correlations between all variables of the study.

**Table 1:** Sample description of the 1993 Pelotas birth cohort members included in this analysis (N = 4,090).

Variable	N	%
**Sex**		
Female	1983	48.5
Male	2107	51.5
**Skin colour**		
White	2630	64.4
Black	577	14.1
Brown	729	17.8
Yellow	74	1.8
Indigenous	79	1.9
**Working outside the home (11 years)**		
No	3906	95.6
Yes	178	4.4
**At least one school failure**	2178	53.3
	**Median**	IQR*
**Family Income (perinatal)** (minimum wages)^#^	2.6	1.5–4.7
**Mother Schooling at birth** (completed studying years)	6.0	4.0–9.0
**Total SDQ Score (11years)**	12.0	7.0–16.0

*IQR: interquartile range

^#^Higher number of missing observations: 74

**Fig 1:**
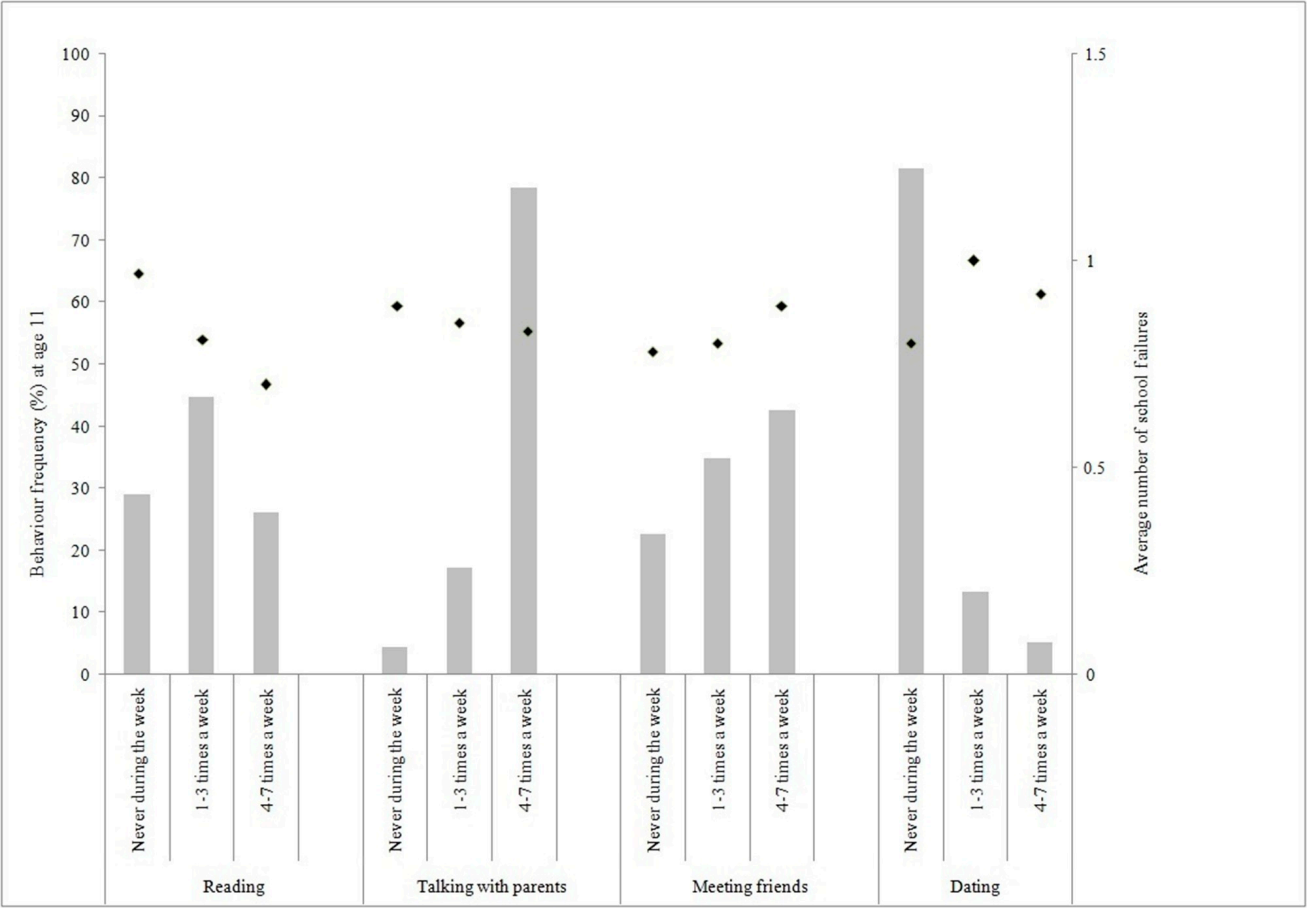
Number of school failures from 11 (reported by parents) to 15 (reported by adolescents) years among adolescents from the 1993 Pelotas Birth Cohort (N = 4,090).

Fig 1 shows leisure-time activity frequencies at 11 years (reading magazines/newspapers/books; talking with parents; meeting friends; dating) and the average number of school failures reported by the adolescents in each of the categories. From the evaluated leisure-time activities at 11 years, the majority of the sample read from once to three times a week (44.7%), talked with their parents four or more days a week (78.5%), met friends four or more times a week (42.6%) and did not date (81.5%). In addition, it was observed that the higher the number of weekdays reading and talking with parents, the lower the average number of failures. Regarding the variable meeting friends, the opposite happened. The dating variable did not show any dose response effect, and the category “from once to three times a week” showed the higher failure average. (Fig 1).

Table 2 shows the crude and adjusted analyses and preventable and etiological fractions of the association between leisure-time activity practice at 11 years and number of school failures from 11 to 15 years. In the crude analysis, statistical significance for all variables was found. The adjusted analysis highlighted an inverse relationship between reading magazines/newspapers/books at 11 years and school failures. Among those who read one to three times a week, the risk of failing was 0.83 (95% CI 0.77 to 0.90) and among those who read four to seven times, this risk was 0.71 (95%CI 0.65 to 0.79) compared to those who did not read anything during the week. Meeting friends in higher frequencies was related to an increased risk of failures. Those who met friends from four to seven times in a week showed a 15% higher risk for failing compared to those who did not meet friends (RR 1.15; 95% CI 1.05 to 1.26). Adolescents in lower levels (1–3 times a week) of dating showed a higher risk of failures [RR = 1.22; (IC95% 1.03 to 1.44)]. The variable talking with parents was not associated with the outcome after adjusting for confounding factors. The preventable fraction for reading showed that around 16% (IC 95%: 11.1; 21.9) of school failures could be avoided if all adolescents had the habit of reading at least once a week.

**Table 2:** Crude and adjusted analyses and attributable risk for leisure-time activities at 11 years and school failure from 11 to 15 years in the 1993 Pelotas birth cohort (N = 4,090).

Activity (days/week)	Crude			Adjusted^#^			Attributable Risk (%)	CI 95%
	RR	IC95%	p-value	RR	IC95%	p-value		
**Reading**							15.9*	11.1–21.9
None	1.00	-		1.00	-			
1–3	0.71	0.65–0.76	<0.001	0.83	0.77–0.90	<0.001		
4–7	0.56	0.51–0.61	<0.001	0.71	0.65–0.79	<0.001		
**Talking with parents**							6.0*	0.0–22.7
None	1.00	-		1.00	-			
1–3	0.85	0.73–1.01	0.061	0.96	0.80–1.14	0.607		
4–7	0.80	0.69–0.93	0.003	0.94	0.80–1.10	0.420		
**Meeting friends**							7.1**	0.5–13.3
None	1.00	-		1.00	-			
1–3	0.98	0.89–1.08	0.689	1.03	0.93–1.14	0.501		
4–7	1.26	1.15–1.38	<0.001	1.15	1.05–1.26	0.003		
**Dating**							4.4**	2.4–6.4
None	1.00	-		1.00	-			
1–3	1.45	1.24–1.71	<0.001	1.22	1.03–1.44	0.022		
4–7	1.16	0.90–1.49	0.262	1.00	0.78–1.30	0.980		

Note: for the calculation of attributable risk, we used at least once a week predictor. P = values correspond to heterogeneity Wald test.

CI 95%: Confidence Interval (α = 0,05); p-values

^#^ Adjustment for mother schooling level and family income (perinatal), work out of the house (at 11 years), gender, skin colour, mental health (at 11years) and number of school failures up to 11 years-old.

*Preventable fraction

**Etiological fraction

The observed and expected prevalence from the 16 possible combinations of the four activities are presented in Table 3. The clusters taking place at higher frequency than expected were the following: only talking with parents showing an observed prevalence 6.77 times higher than expected, and talking with parents and the habit of reading showing an observed prevalence 6.15 times higher than expected. Other clusters could be observed but of a lower magnitude.

**Table 3:** Prevalence of leisure-time activity clusters at 11 years among adolescents from the 1993 Pelotas Birth Cohort.

	Leisure-Time activities at 11 years				Observed (%)	Expected (%)	O/E*
	Reading	Talking with parents	Meeting friends	Dating			
4	+	+	+	+	11.00	9.59	**1.15**
3	+	+	+	-	42.40	41.68	**1.02**
	+	+	-	+	1.26	0.49	**2.55**
	+	-	+	+	0.36	2.88	0.13
	-	+	+	+	4.82	4.09	**1.18**
2	+	+	-	-	13.20	2.15	**6.15**
	+	-	-	+	0.11	0.15	0.74
	+	-	+	-	1.13	12.52	0.09
	-	-	+	+	0.38	1.23	0.31
	-	+	+	-	15.70	17.78	0.88
	-	+	-	+	0.68	0.21	**3.23**
1	+	-	-	-	0.74	0.65	**1.15**
	-	+	-	-	6.20	0.92	**6.77**
	-	-	+	-	1.17	5.34	0.22
	-	-	-	+	0.11	0.06	**1.74**
0	-	-	-	-	0.83	0.28	**3.02**

*O: observed; E: expected. In bold, those behaviours that occurred more than the expected proportion

+ Present Factor;−Absent Factor.

**Fig 2:**
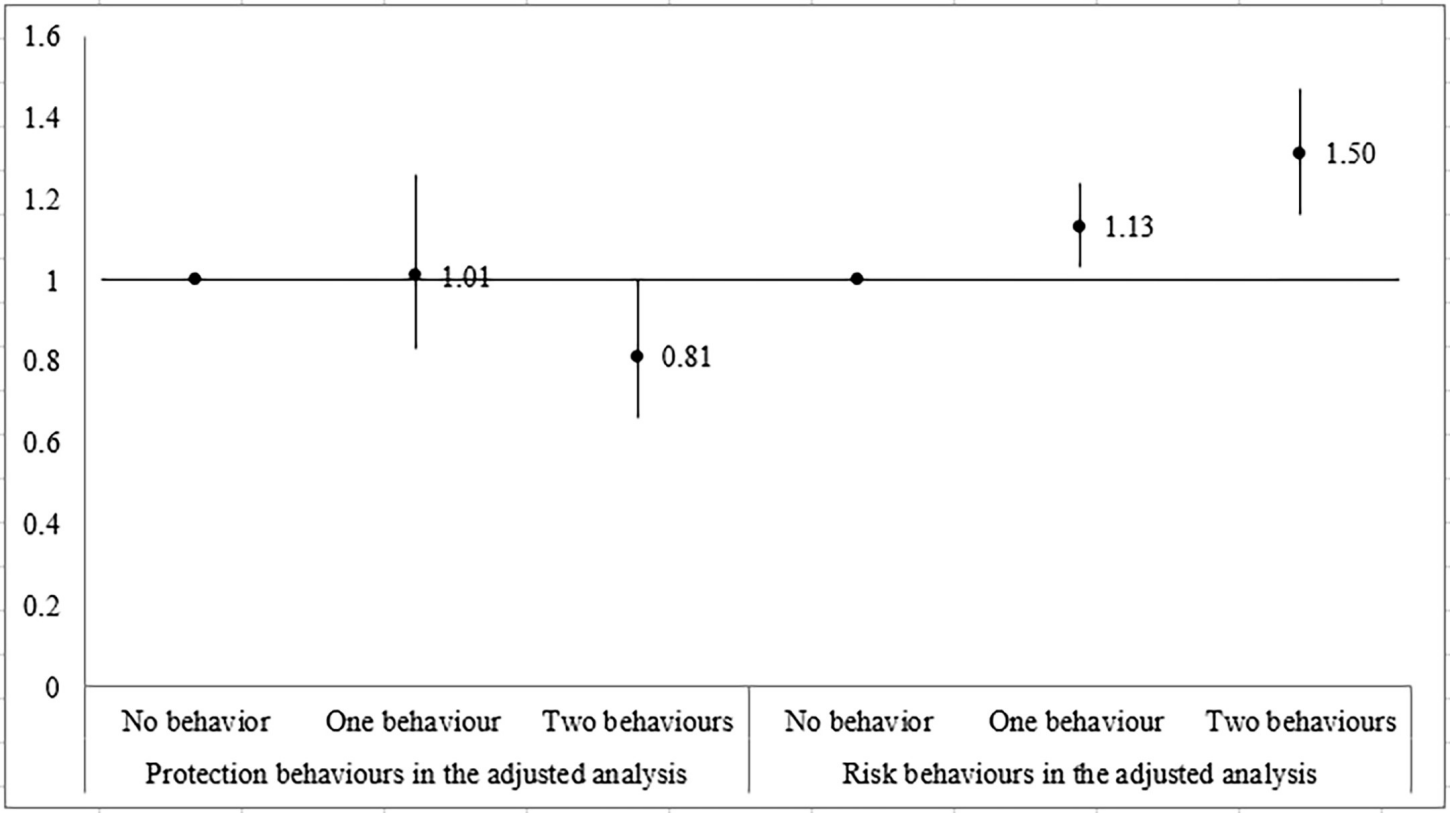
Association between number of protection or risk behaviors and school failure in adolescents from the 1993 Pelotas Birth Cohort (N = 4,090).

Fig 2 shows the association between the numbers of leisure-time activities which were either protection or risk factors for the outcome in the crude analysis. It may be observed that the higher the number of behaviours considered protectors in the crude analysis, the lower school failure was (p<0.001). However, school failure was higher (p<0.001) with the increase in the number of “risky” leisure-time activities in the crude analysis.

## Discussion

In the present study the association between different leisure-time activities and school failure in adolescents was assessed. More than half of the adolescents failed at least once from 11 to 15 years. Those who carried out reading activities showed a lower number of failures while those who dated and went out with friends showed higher numbers. Talking with parents was not associated with the number of school failures in the period.

The prevalence of at least one school failure from 11 to 15 years found in this study was 53.3% which is higher than the percentage observed in studies conducted in developed countries such as France (14.8%) and Spain (18.9%) [12,25]. This difference may be explained due to several aspects concerning the Brazilian reality such as low quality teaching [26] and infrastructure [27], lack of human resources [28], professional depreciation [28,29] and management problems [30]. These difficulties in the educational process may influence adolescents negatively at a great extent, promoting lack of interest in studying. Sposito (2008), for instance, highlights a paradox: students understand the need for education in order to build a better future, but they do not give immediate importance to school [31].

The findings between reading activities at leisure-time and the number of school failures are in agreement with previous studies. Regardless the socioeconomic and demographic characteristics, adolescents who are engaged in more frequent reading activities not only show more interest in their school life but also find school tasks easier to deal with [14,15]. Wigfield & Guthrie (1997) highlight the importance of collaboration between school and family in order to encourage reading habits due to its large contribution to a good school performance [13]. In this study, the impact measure showed the importance of this activity in adolescent’s school performance.

Although the present study has not found any association between school failure and talking with parents, one believes that emotional bonding and family support help adolescents to face their problems due to poor school performance [14]. Studies have shown that students bearing more than one behaviour problem (stealing, lying, fighting, among others) and with learning difficulties (being left behind at school and difficulty in writing) were less involved in school activities, less supported by parents and were less involved in family activities [15,32]. Jeynes (2010) showed the importance of communication, especially in the relationship between parents and adolescents. The author highlights the importance of a positive and frequent communication between parents and their offspring to achieve the success in school [33]. For Latin-american families, communication among members seems to be crucial. A study with 40 Latino families living in United States has shown this fact and the importance of parent-child communication in all dimensions of school activities [34]. The result found in this study may be due to lack of an investigation regarding the quality of the conversation with parents and therefore overestimating the prevalence of this activity. Almost all adolescents reported talking to their parents at leisure-time. Thus, the effect measure could tend to nullity, as observed in our study, as lower variability in the exposure occurred.

Regarding meeting friends, literature shows that outside support may help adolescents’ school development [35]. On the other hand, this study found increased levels of this activity associated to a higher number of school failures. One of the possible explanations for this finding may be the categorization for the exposure variables: going out with friends “never during the week” and “one day or more in the week”. Meeting friends too many times may put strain on consolidating a study routine as stated by Amparo et al. (2008) [14]. However, the above studies do not question if the interest in meeting friends is either a cause or consequence from a certain lack of interest in school and in the educational process. Our analysis showed the same pattern of association for meeting friends, but the relationship is non-monotonic and the intent of the meetings was not recorded.

Low levels of dating have shown to increase the number of school failures. It is relevant to highlight that this variable was collected at 11 years and it may be considered a negative behaviour due to its early occurrence [36]. In addition, dating in early ages may lead adolescents to prioritize the new relationship instead of their commitment to school. Such fact has raised important questions in order to understand this association, such as, parents not being aware of their children’s leisure-time activities. The 2012 Brazilian National Survey of Health at School (PeNSE) identified that 41.5% of the parents were not aware of their children’s activities. Another aspect raised in some studies is the fact that adolescents bearing a harmful behaviour to health (drinking and/or smoking, using drugs, unsafe sex, among others) tend to have this behavioural pattern along their lives different from those who do not have these habits [37,38]. There are studies showing that adolescents who are dating may develop risky behaviours which interfere with school performance, such as frequent drinking, leading to school failure [16,17]. This body of evidence, which deserves a more thorough investigation, suggest that dating at 11 years may act as a marker for a behaviour pattern.

Based on the cluster analysis, it was observed that only talking with parents, or having reading habits and talking with parents were behaviours which occurred six times more often than expected. It is important to highlight that these clusters are made up by leisure-time activities considered socially positive [37]. It is likely that adolescents, who talk with his/her parents more frequently and live in a more harmonious environment, are more encouraged and supported by their parents to achieve school success in comparison to those who do not show these characteristics [14]. In this study, the higher the number of leisure-time activities which could be considered as “good” behaviours in the crude analysis, the lower the number of school failures, different from those leisure-time activities considered as risk markers for the outcome in the crude analysis.

Within the limitations of this study, the difference between the methods to assess school failure in the two follow-ups is highlighted which may underestimate the results due to the following reasons: (i) two ways for checking school failure. At 11 years there was a more thorough description for school failure, evaluating it in each year of primary school and at 15 the considered school failure was “once in a life time”, a choice which may have underestimated the number of school failures; (ii) the information source. The questions were answered by the adolescents’ mother at 11 years and by the adolescents themselves at 15 years. Both parents and adolescents may have been embarrassed to admit school failure, as this fact is a social burden, and therefore underestimating the number of failures. Nevertheless, these limitations tend to take the effect measure to no statistical association. This paper has not assessed the relationship between physical activity and school failure as a different study using the same cohort had previously been published showing that individuals with higher amounts of physical activity (> 1,000 minutes per week) at leisure-time experienced more school failures in comparison to their counterparts [11].

Among the positive aspects, the high response rate is highlighted in both follow-ups and the similarity between the samples of this study when compared to the original cohort (data not shown). It should also be noted that due to the type of study design, the possibility of reverse causality is minimized. Moreover, this study attempts to fill a gap in the literature about the theme and it is the first one in Brazil, to the best of the authors’ knowledge, to investigate the relationship between school failure and leisure-time activities during adolescence.

## Conclusion

In conclusion, the context in which the adolescent is inserted plays a relevant role in their academic performance. It is of outmost importance that schools, as an educational institutions, participate actively, along with family support, in projects to encourage leisure-time activities such as reading. In this study, we have shown that relative simple leisure-time activities such as reading could reduce the number of school failures, even in low frequency during the week. The relationship between doing leisure-time activities and school failure is very complex, and yet more studies are needed to further investigate the topic, using different methodologies, showing how and which other factors may influence the associations. To decompose leisure-time activities could, in more specific activities, help to understand these complex relationships. This will aid in the development of actions targeted to reduce the levels of school failure even further.

## Supporting information

S1 Table Sup Table 1. (DOCX)

S1 Data Data school failure. (XLS)
