# Supplementary material for: Association between leisure-time activities and school failure in adolescents: The 1993 Birth Cohort
Source: PLoS One. 2018 Nov 7;13(11):e0205793. doi: 10.1371/journal.pone.0205793 (PMC6221260; doi:10.1371/journal.pone.0205793)
Supplement: S1 Table — (DOCX) [file pone.0205793.s001.docx]

Supplementary Table 1 – Correlations between all variables of the study. The Pelotas 1993 Birth Cohort.

|  | **School failure from 11 to 15 years** | **Reading** | **Talking with parents** | **Meeting friends** | **Dating** | **Gender** | **Mother schooling level** | **Family income** | **Work out of the house** | **Skin colour** | **Mental health** | **Number of school failures at 11 years** | **Number of school failures at 15 years** |
| --- | --- | --- | --- | --- | --- | --- | --- | --- | --- | --- | --- | --- | --- |
| School failure from 11 to 15 years | 1.00 |  |  |  |  |  |  |  |  |  |  |  |  |
| Reading | -0.24 | 1.00 |  |  |  |  |  |  |  |  |  |  |  |
| Talking with parents | -0.07 | 0.16 | 1.00 |  |  |  |  |  |  |  |  |  |  |
| Meeting friends | 0.12 | -0.03 | 0.13 | 1.00 |  |  |  |  |  |  |  |  |  |
| Dating | 0.20 | -0.08 | -0.02 | 0.27 | 1.00 |  |  |  |  |  |  |  |  |
| Gender | -0.19 | 0.16 | 0.04 | -0.21 | -0.59 | 1.00 |  |  |  |  |  |  |  |
| Mother schooling | -0.32 | 0.18 | 0.04 | -0.04 | -0.02 | -0.04 | 1.00 |  |  |  |  |  |  |
| Family income | -0.33 | 0.13 | 0.07 | -0.04 | -0.03 | -0.01 | 0.47 | 1.00 |  |  |  |  |  |
| Work out of the house | 0.16 | -0.05 | -0.01 | 0.16 | 0.15 | -0.18 | -0.16 | -0.25 | 1.00 |  |  |  |  |
| Skin colour | 0.17 | -0.07 | -0.07 | 0.05 | 0.04 | 0.00 | -0.22 | -0.29 | 0.03 | 1.00 |  |  |  |
| Mental health | 0.24 | -0.18 | -0.13 | 0.05 | 0.11 | -0.08 | -0.18* | -0.12* | 0.17 | 0.14 | 1.00* |  |  |
| School failures at 11y | 0.28 | -0.28 | -0.08 | 0.14 | 0.15 | -0.19 | -0.33* | -0.18* | 0.18 | 0.26 | 0.26* | 1.00* |  |
| School failures at 15y | 0.83 | -0.30 | -0.08 | 0.14 | 0.19 | -0.21 | -0.39* | -0.24* | 0.18 | 0.23 | 0.31* | 0.73* | 1.00* |

Note: correlation coefficients were obtained through polychoric correlation

* Pearson’s correlation coefficient.
